# Insight Into the Role of Autophagy in Osteosarcoma and Its Therapeutic Implication

**DOI:** 10.3389/fonc.2019.01232

**Published:** 2019-11-15

**Authors:** Jianfang Niu, Taiqiang Yan, Wei Guo, Wei Wang, Zhiqing Zhao

**Affiliations:** ^1^Musculoskeletal Tumor Center, Peking University People's Hospital, Beijing, China; ^2^Beijing Key Laboratory of Musculoskeletal Tumor, Beijing, China

**Keywords:** osteosarcoma, autophagy, PI3K/AKT/mTOR pathway, ROS/JNK pathway, hypoxia, cancer therapy

## Abstract

Osteosarcoma is an aggressive bone cancer that frequently metastasizes to the lungs. The cytotoxicity of most chemotherapeutics and targeted drugs in the treatment of osteosarcoma is partially lessened. Furthermore, there is a poor response to current chemo- and radiotherapy for both primary lesions and pulmonary metastases of osteosarcoma. There is a clear need to explore promising drug candidates that could improve the efficacy of osteosarcoma treatment. Autophagy, a dynamic and highly conserved catabolic process, has dual roles in promoting cell survival as well as cell death. The role of autophagy has been investigated extensively in different tumor types, and a growing body of research has highlighted the potential value of using autophagy in clinical therapy. Here, we address significant aspects of autophagy in osteosarcoma, including its functions, modulation, and possible therapeutic applications.

## Introduction

Osteosarcoma (OS) is the most prevalent primary malignant bone neoplasm in children and adolescents (1). In most cases, OS originates from the long bone around the knee joint (2) and tends to metastasize to the lung. Before the emergence of chemotherapy in the 1970s, limb OS was mainly treated by amputation. Not only did the patients suffer from limb loss or disfigurement and significant psychological trauma, but 80% of the patients still inevitably died of lung metastasis. The 5-year survival rate of OS has been greatly improved, from <20 to 50–60%, owing to the use of neoadjuvant chemotherapy in combination with surgery. For patients with early lung metastasis, the 5-years survival rate is <20%. However, 20% of patients without early lung metastasis either relapse locally or have eventual pulmonary metastasis (3). In the past 30 years, there has been no major breakthrough in the treatment of OS, and the overall survival rate has reached a plateau. So far, the poor response of OS to chemotherapy is the primary challenge, and new pharmacological molecules or strategies need to be explored to further improve OS patients' survival, those who are currently insensitive to chemotherapy regimens.

**Figure 1:**
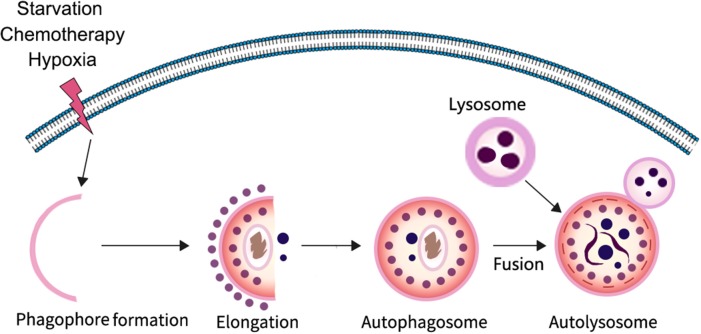
The progress of autophagy. In response to various stressors such as starvation and hypoxia, a multi-step autophagy is activated for the cell to survive the microenvironment.

Autophagy is a highly conserved catabolic process in which parts of unwanted proteins or damaged organelles are encapsulated in a bilayer membrane vesicle and delivered to the lysosome for degradation (4–7). It is particularly important for maintaining homeostasis and cell survival in response to nutrient deficiency. The process of autophagy involves three major steps: formation and elongation of the phagophore (isolation membrane), double-membrane autophagosome formation, and formation of an autolysosome by fusion of autophagosomes with lysosomes (8). There are three different types of autophagy identified based on the way in which intracellular substrates are transported to lysosomes, including macroautophagy, microautophagy, and chaperone-mediated autophagy. In the process of macroautophagy, it is the isolation membrane that encapsulates the degradable substance in the form of vesicles, whereas in microautophagy the lysosomal membrane forms itself into vesicles encapsulating the substrate in the cytoplasm. In contrast to other forms of autophagy, chaperone-mediated autophagy does not form any membrane-coated vesicles, but rather transports substrate proteins through lysosomal membranes. At present, macroautophagy is the most widely studied; therefore, in this review we mainly focus on macroautophagy. In the typical process of macroautophagy small membrane vesicles (phagocytic vesicles) first appear in the cytoplasm, extend to form cup-like structures, and then close to form early autophagosome (AVi) enclosed by either a membrane bilayer or multi-layered membranes; autophagosomes then fuse with lysosomes to form degradable autophagic vesicles known as autolysosome (AVd), whose inner membrane and contents are degraded by lysosomal hydrolases (Figure 1).

Autophagy is a highly regulated process involving several key mediators. Several autophagy-related (Atg) genes have been identified that participate in this process. *ATG1*, a serine/threonine kinase, was the first *ATG* gene identified as necessary for autophagy. The first step is the formation of a complex including ATG1, ATG13, and ATG17, after which ATG9 adds to the developing phagophore. Class III phosphatidylinositol-3 kinase (PI-3KIII), vesicular protein sorting 34 (VPS34), and beclin 1 also contribute to this process (9). Beclin 1 is a mammalian-specific autophagy-related gene (Atg), homologous to *ATG6*, which plays an important role in autophagy and the development of tumors (10). The combination of ATG12 and ATG5 promotes the elongation of the phagophore. It is also aided by ATG7 and ATG10 (8). ATG8, a key mediator in autophagy, localizes to the autophagosome and its membrane. The conjugation of ATG8 with phosphatidylethanolamine (PE) is also involved in elongation of the phagophore, and ATG12 is indispensable for the formation of ATG8-PE. An additional protein found to participate in autophagy regulation is cytosolic microtubule-associated protein 1 light chain 3 (LC3). In autophagosome formation, LC3, the earliest autophagic marker, is in an unprocessed form and can be converted to a proteolytic form, LC3-I. When autophagy occurs, a ubiquitin-like modification transforms LC3-I into LC3-II, which then translocates from the cytoplasm to the autophagosome membrane (11–13). The amount of LC3-II or the ratio of LC3-II/LC3-I is positively correlated with the number of autophagosomes, which could reflect the autophagic activity of cells. In addition, equestosome 1 (P62/SQSTMl) is a stress-induced intracellular protein that acts as a multifunctional protein for selective autophagy. It participates in many signal transduction pathways, regulates the expression of cell proliferation genes, as well as those involved in oxidative stress and autophagy. The expression level of p62 is inversely proportional to autophagic activity, and it can be used as an auxiliary index to detect autophagy (14). In the final stage of autophagic activation, the autophagosome fuses to the lysosome, thereby forming the mature autolysosome. The membranes and the contents of the autophagosome are degraded by lysosomal hydrolases. The autolysosome is broken down and degradation products, such as amino acids, are transported back to the cytoplasm. These final products can be used for material synthesis or can be processed to provide cells with energy.

Cancer is the first disease found to be associated with autophagy. In 1998, Liang et al. (15) discovered the BCL2 binding protein beclin 1, a mammalian homolog of yeast *ATG6*. In 2003, they further demonstrated that beclin 1 had tumor-suppressing properties (16). Beclin 1 is located on human chromosome 17q21 and has a single allele deletion in 40–75% of breast, ovarian, and prostate tumors (17–19). CSN3 (subunit 3 of the COP9 signalosome) can activate autophagy by maintaining high expression levels of beclin1, and autophagy can in turn promote lung metastasis in osteosarcoma (20). In the tumor microenvironment, various conditions can induce autophagy including hypoxia (21), starvation (22), cytokines (23), and chemotherapy (24). During the last decade, autophagy, which can serve as a source of cellular energy, has been recognized to promote cell survival by providing cells with amino acids by eliminating old or damaged organelles (9, 11). However, autophagy is still generally thought of as a type of cell death, along with apoptosis and necrosis (25). At present, there is no complete and detailed account of autophagy in OS; therefore, in this review, we will explore the different roles of autophagy in OS and further discuss the prospect of harnessing autophagy as an antineoplastic therapy.

## Regulation of Autophagy in Response to Multiple Stressors

**Figure 2:**
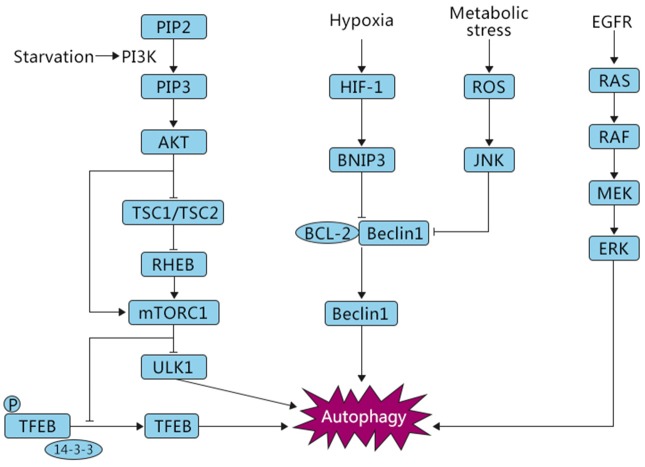
Autophagic pathways common in OS. Under different stress conditions, i.e., starvation and hypoxia, autophagy is triggered by signaling cascades including mTOR, HIF-1, and ROS/JNK, which play opposite roles in the induction of this process. The inhibition of autophagy is mTOR-dependent, whereas oxygen deficiency and ROS signaling are associated with activation of autophagy, eventually leading to either survival or death of OS cells.

Several studies have reported that autophagy is involved in both oncogenic and tumor-suppressing activities in response to cellular stressors (26), and various molecular mechanisms have been reported to regulate autophagy (Figure 2). Nutritional deficiency can lead to cell growth inhibition, which is partially achieved by relying on the mammalian target of rapamycin (mTOR) signaling pathway. The mTOR signaling pathway can respond to the intracellular levels of amino acids, energy, and oxygen. mTOR kinase is downstream of phosphoinositide-3-kinase (PI3K) and protein kinase B (AKT) and negatively regulates autophagy (27). There are two key factors, mTORC1 and mTORC2, involved in the mTOR pathway. Several studies have shown that mTORC1 plays a critical role in negatively regulating autophagy (28). In normal conditions, UNC-51-like kinase 1 (ULK1), which can promote autophagy, is suppressed by mTORC1. Furthermore, mTORC1 phosphorylates ATG13, a pro-autophagic molecule that positively regulates ULK1, and directly inhibits its expression (29). AKT, a key mediator involved in the regulation of many signaling pathways, mediates the suppression of autophagy. PI3K stimulates the production of PIP3, which in turn phosphorylates AKT. Subsequently, the phosphorylated AKT catalyzes mTORC1, eventually leading to the inhibition of autophagy. In addition, AKT can also block TSC1/TSC2, resulting in the production of RHEB, which then phosphorylates mTORC1, causing the inhibition of autophagy. Normally, when the PI3K/AKT/mTOR pathway is activated, autophagy is inhibited and cells are in a normal state of proliferation (12, 30). Conversely, when cells are starved or hypoxic, mTOR is suppressed and autophagy is activated (27).

Autophagy can also be induced by other metabolic stressors. Reactive oxygen species (ROS) are a toxic byproduct of cell metabolism. A small amount of ROS can promote cell proliferation and differentiation, but excessive ROS can result in significant damage to cell structures (31, 32). Mitochondria, the main sites of oxidative respiration of cells, are one of the important sources of ROS. There are a series of complex signal transductions and interactions between ROS and the mediators of autophagy that regulate the response to cellular stress. Autophagy is often accompanied by changes in the level of ROS. Excessive ROS can also damage mitochondria, leading to mitophagy, which is a specific selection process. A study found that parthenolide, an anti-inflammatory and anti-tumor substance, mediated the occurrence of mitophagy, as evidenced by increased PINK1 and parkin translocation to mitochondria and enhanced conversion of LC3-I to LC3-II, which induced autophagic death of OS cells by stimulating the activation of ROS (33). c-Jun N-terminal kinase (JNK) is an important member of the MAPK protein family, which is involved in programmed cell death. ROS have been reported to act as key signaling molecules that induce the activation of MAPK family members, including JNK (34). Activated JNK inhibits BCL2 combined with beclin 1, thus promoting the expression of beclin 1 and autophagy activation.

In addition, recent evidence has shown that the transcription factor TFEB, belonging to the MITF/TFE family, participates in the modulation of autophagy in response to cellular stressors. Settembre et al. found that overexpression of TFEB can enhance autophagy, and conversely, suppressed TFEB expression downregulates autophagy (35). The cellular localization of TFEB was discovered to be regulated by mTORC1. Under nutrient-rich conditions, V-ATPase, constitutively active (GTP-bound) RAG GTPase, and RAG GTPase regulatory factors can form a complex, recruit mTORC1 to the lysosomal membrane, and activate it. Activated mTORC1 phosphorylates TFEB on serine 142 and 211. This phosphorylation promotes the binding of TFEB to the chaperone-like cytosolic protein 14–3–3, resulting in the retention of TFEB in the cytoplasm. In contrast, in starvation conditions, inactivated mTORC1 cannot phosphorylate TFEB, promoting the dissociation of TFEB from the complex and translocation of TFEB from the cytoplasm to the nucleus (36, 37). In the nucleus, TFEB targets a variety of genes involved in autophagy and metabolism to promote cell survival (38). Wu et al. have shown that arsenic trioxide (ATO), an anti-carcinogenic chemical, induced TFEB dephosphorylation on serine 142 and promoted TFEB nuclear translocation, which resulted in the activation of autophagy, and autophagic cell death in OS (39). The epidermal growth factor receptor (EGFR) is also a key regulator during autophagy activation. A study showed that the RAS/RAF/MEK signaling, an EGFR-depended pathway, is also involved in autophagy activation (40). Overall, autophagy is a multistep and complex physiological process that involves multiple signals that enable the cell to survive microenvironmental stresses.

## The Regulation of Autophagy in OS

### PI3K/AKT/mTOR Pathway

A recent study suggested that TSSC3 enhances autophagy through inactivation of the Src-mediated PI3K/AKT/mTOR pathway and inhibits proliferation, invasion, and migration of OS cells (41). However, a natural compound from medicinal herbs, deoxypodophyllotoxin (DPT), was found to induce cytoprotective autophagy via inhibition of the PI3K/AKT/mTOR pathway in OS U2OS cells. Furthermore, inhibition of autophagy using 3-methyladeine (3-MA) accelerated the apoptotic response promoted by DPT (42). A link between mTOR and autophagy has also been documented by Horie et al. They showed that a combination of inhibitors of mTOR and autophagy could enhance the apoptotic ratio of OS MG63 cells (43). Heat shock protein 90 (HSP90) is highly expressed in tumor cells, suggesting that it may play an important role in the survival and malignant behaviors of tumors. The HSP90 inhibitor geldanamycin (GA) has been reported to induce autophagy through inhibition of mTOR. GA alone promotes cell apoptosis, but when combined with the autophagy inhibitor 3-MA, apoptosis is greatly increased. The above results suggested that GA-induced autophagy plays a cytoprotective role (44). Similarly, one of the factors in the HSP90 family, HSP90AA1, has also been reported to function in tumor progression (45). A significant enhancement of HSP90AA1 at both the transcriptional and post-transcriptional levels was observed after treating OS cells with cisplatin (CDP), doxorubicin (Dox), and methotrexate (HD-MTX). To explore the potential role of chemotherapy-induced HSP90AA1 in the drug sensitivity of OS cells, OS cells transfected with *HSP90AA1* shRNA were established. The authors found that knockdown of *HSP90AA1* suppressed cell proliferation and increased the sensitivity to CDP, Dox, and HD-MTX. Furthermore, they demonstrated that HSP90AA1 promotes autophagy in OS cells treated with chemotherapeutic agents, as evidenced by elevated expression of LC3-II, a decreased amount of P62, and the formation of autophagosome-lysosomes. In addition, they further proved that HSP90AA1 induces autophagy by impairing the PI3K/AKT/mTOR pathway. More importantly, the autophagy inhibitor 3-MA could efficiently decrease the proliferation rate, indicating that inhibition of autophagy reversed the HSP90AA1-mediated cytoprotection against chemotherapy. The above results indicate that autophagy is required for HSP90AA1-induced proliferation and survival of OS cells (46). Overall, targeting autophagy is a promising approach that requires further investigation. Importantly, mTOR inhibitors, e.g., rapamycin, ridaforolimus, and LY3023414 are being studied in clinical trials involving a range of tumor types. Results from such trials will yield valuable information regarding the safety of these pharmacological compounds and their anti-tumor efficacy.

### ROS/JNK Pathway

In addition to the PI3K/AKT/mTOR pathway, ROS/JNK signaling is also a key regulator of OS autophagy. Erianin, a natural compound derived from *Dendrobium chrysotoxum*, has been demonstrated to induce autophagy by activating ROS/JNK signaling cascades and inhibiting the proliferation of OS cells (47). Studies have shown that JNK activation can induce apoptosis (48) and autophagy (47) to a significant degree. Undoubtedly, autophagy is usually accompanied by the induction of apoptosis. Ma et al. have reported that cinobufagin could simultaneously trigger apoptosis and autophagy via activation of the ROS/JNK/P38 axis (49). Another report by Li et al. (50) reported that inhibition of celastrol-induced autophagy diminished caspase-dependent apoptosis. Arsenic sulfide (As_2_S_2_), the main ingredient of realgar, was reported to have anti-cancer activity in several tumor types, including gastric cancer and colon cancer. Wang et al. found that As_2_S_2_ has dose- and time-dependent anti-proliferative activity in OS cells. Furthermore, they showed that As_2_S_2_ could promote autophagy and that treatment with 3-MA could reinforce the inhibitory effect of As_2_S_2_ on OS cells. They further found that As_2_S_2_ enhanced ROS generation, phosphorylated JNK, and that pretreatment with the ROS scavenger N-acetyl-L-cysteine (NAC) could significantly decrease the levels of ROS, JNK, and autophagy-related proteins. More importantly, NAC and the JNK inhibitor SP600125 increased the effect of As_2_S_2_ on OS cells. In addition, NAC and SP600125 had a significant inhibitory effect on As_2_S_2_-mediated G2/M phase arrest as shown by flow cytometric assay. The above findings suggest a mechanism of cellular protection through autophagy induced by As_2_S_2_ through activation of the ROS/JNK signaling pathway (51). Interestingly, the antibiotic salinomycin was also observed to induce autophagy as shown by increased expression of autophagy-related proteins and accumulation of acidic vesicular organelles (AVO). Subsequently, treatment with NAC suppressed salinomycin-stimulated autophagy owing to reduced LC3II expression and AVO accumulation, demonstrating that ROS functions as an upstream regulator in salinomycin-induced autophagy in OS cells. Furthermore, salinomycin in combination with 3-MA enhanced salinomycin-provoked apoptosis of OS cells when compared with salinomycin alone. Taken together, these results showed that salinomycin-induced autophagy, as a tumor survival mechanism, might be a potential target in OS therapy (52). Conversely, andrographolide (AG) induces a cytotoxic autophagy response as reported by Liu et al. AG enhanced the levels of autophagy-regulated proteins such as LC3II, beclin 1, and ATG5 and could also induce a massive autophagy flux, with the increase of autophagosomes and autolysosomes in OS cells. Furthermore, knockdown of beclin 1 by siRNA clearly increased the viability of AG-treated OS cells. Treatment with AG phosphorylated JNK, and the specific JNK inhibitor SP600125 visibly reduced the expression of autophagy-related proteins. These findings suggest that the eventual cell death resulted from the induction of JNK-associated autophagy (53). Altogether, the ROS/JNK pathway is crucially involved in the regulation of autophagy by various factors. Identifying the anti-oncogenic effects of these compounds in OS may provide some clues for further development of clinical applications.

### MicroRNAs

MicroRNAs (miRNA) are a class of endogenous non-coding small RNAs about 22–24 nucleotides in length that regulate gene expression at the post-transcriptional level (54). Studies have shown that miRNAs regulate many cellular processes, including cell growth, differentiation, inflammation, and apoptosis (55, 56). Emerging evidence demonstrates that miRNAs are associated with the regulation of autophagy and play an important role in the resistance or sensitivity to chemotherapy (57–59).

Exposure to CDP has been found to significantly decrease the levels of miR-199a-5p and induce the activation of autophagy. Induction of miR-199a-5p expression in OS inhibited CDP-mediated autophagy and reinforced proliferation inhibition. In addition, beclin 1, a key mediator of autophagy, is a direct target of miR-199a-5p, suggesting that miR-199a-5p may inhibit CDP-induced autophagy by targeting beclin 1 expression (60). Upregulation of miR-140-5p was also observed in OS treated with chemotherapy. Further research showed that upregulation of miR-140-5p enhanced anti-cancer drug-induced autophagy and alleviated the chemotherapy-mediated decrease in cell proliferation and viability by regulating inositol 1,4,5- trisphosphate kinase 2 (IP3K2) signaling (61). Xu et al. found that miR-30a, a member of the miR-30 family, was significantly reduced in Dox-resistant OS cells, whereas increased autophagy was observed as indicated by elevated expression of beclin 1 and conversion of LC3 from LC3-I to LC3-II. More importantly, overexpression of miR-30a clearly enhanced Dox-stimulated apoptosis and inhibited autophagy. A luciferase reporter assay further showed that miR-30a binds directly to the 3'-UTR of beclin 1, indicating that miR-30a caused drug insensitivity by inhibiting beclin 1-induced autophagy (62). The role of miR-30a has also been studied in other tumors. The inhibition of miR-30 was found to activate autophagy and increase chemoresistance in response to imatinib treatment in chronic myeloid leukemia (63). Chang et al. found that autophagy was activated in OS cells treated with Dox as evidenced by elevated expression of LC3-II and ATG5. The authors further showed that miR-101 mimics significantly reduced the doxorubicin-induced formation of AVO, the conversion of LC3-I to LC3-II, and the expression of ATG4, whereas there was little effect on ATG5 expression. Importantly, an MTT assay indicated that overexpression of miR-101 could accelerate the Dox-mediated viability decrease in OS cells (64). The associations among miRNA, autophagy, and chemoresistance are rather complicated and many of these interactions have not been clarified, but the involvement of miRNA in chemotherapy-induced autophagy gives us a more comprehensive understanding of the mechanism of drug resistance and provides a new direction for the emergence of novel antineoplastic drugs.

## Hypoxia-induced Autophagy in OS Progression

Rapid proliferation of cancer cells often results in oxygen deprivation in the tumor microenvironment because of the limited blood supply. Hypoxia, a common phenomenon in most solid tumors, can induce a dramatic enhancement of autophagy (65, 66). For instance, Rahim et al. (67) focused on 98 specific regulators of autophagy under hypoxic conditions. They found that ATG9A was commonly upregulated in glioblastoma (GBM) in conditions of both short-term and long-term hypoxia. Furthermore, increased autophagy was also observed by enhanced conversion of LC3-I to LC3-II under hypoxia. In addition, interfering with ATG9A expression dramatically blocked GBM cell proliferation and tumor growth *in vivo* (67). In T-cell lymphoma, autophagy was shown to be increased when cells were incubated in hypoxic conditions. Hypoxia-mediated autophagy also clearly decreased the chemosensitivity of lymphoma cells treated with Dox (68).

In OS, several studies have demonstrated a relationship among hypoxia, autophagy, and tumor progression. Guo et al. showed that paclitaxel, derived from the bark of the yew tree, was able to trigger a protective autophagy response via increased expression of beclin 1 and LC3II/I. Hypoxia- inducible factor 1 (HIF1), a key nuclear transcription factor regulating gene expression in response to hypoxia, was reported to be associated with tumor progression, resistance to chemotherapy and radiotherapy (69–72), and activation of autophagy (73, 74). Adenovirus E1B 19 kDa interacting protein 3 (BNIP3), which functions downstream of HIF1, competes with beclin 1 to bind BCL2, resulting in the release of beclin 1 from the beclin 1-BCL2 complex and the induction of autophagy (Figure 2). Pre-treatment of OS cells with paclitaxel enhanced the levels of HIF-1α. Interestingly, the upregulated autophagy-associated protein expression was partly attenuated in paclitaxel-treated OS in the presence of the HIF-1α inhibitor YC-1, indicating that additional mechanisms are involved in paclitaxel-mediated modulation of autophagy. Co-treatment with YC-1 augmented the suppressive effects of paclitaxel (75). Similarly, another recent report suggested a potential tumor-promoting function of hypoxia-induced autophagy by conferring radioresistance to OS cells. The authors further discovered that the autophagy inhibitor chloroquine (CQ) and 3-MA both significantly decreased the survival rate of OS cells under hypoxia following irradiation treatment (76), implying a possible novel strategy to overcome radioresistance in OS. At present, there are few studies on the relationship between hypoxia and autophagy in OS. More research is needed to comprehensively understand the role of hypoxia-induced autophagy in OS.

## Other Regulatory Mechanisms in OS

A number of other mechanisms have been found to regulate autophagy. For example, the overexpression of Wilms tumor suppressor 1 (WT1), a transcription factor with a large number of target genes, positively correlates with the activation of autophagy in OS. The AKT/JNK pathway was found to be involved in the active autophagy mediated by WT1, as autophagy was blocked by the JNK inhibitor SP600125 or the AKT inhibitor MK-2206 in cells in which WT1 was overexpressed. Interestingly, the expression of arrest-specific 1 (GAS1) was required for WT1-regulated autophagy via the AKT/JNK signaling pathway (77). Matrine, one of the main components of the traditional Chinese medicine *Sophora flavescens*, was reported to inhibit tumors including hepatoma and leukemia (78, 79). Ma et al. have revealed that matrine also exerts an anti-cancer effect in OS, as it inhibited OS cell proliferation in a dose- and time-dependent manner. Moreover, the authors found that treatment with matrine stimulated the activation of autophagy by measuring the conversion from LC3-I to LC3-II and the accumulation puncta of GFP-LC3. They further confirmed that matrine-induced autophagy was ERK dependent. Matrine treatment increased the level of p-ERK but not t-ERK, and further pre-treatment with U0126, an ERK inhibitor, could decrease the expression of p-ERK and the ratio of LC3II/LC3I, demonstrating that ERK activation was involved in matrine-regulated autophagy. In addition, inhibition of autophagy by CQ enhanced the cytotoxicity of matrine, indicating that matrine-induced autophagy played a cytoprotective role in OS (80). Another recent study investigated the role of Wnt/β-catenin signaling in the induction of autophagy in OS. Overexpression of *BECN1* could reduce the cytotoxicity of gemcitabine against OS, which could be reversed by activating the Wnt/β-catenin pathway. The Wnt/β-catenin pathway was found to increase the sensitivity of beclin 1-overexpressed OS cells to gemcitabine-induced cell death by downregulating the expression of beclin 1 (81). Surprisingly, KP46, a bone-targeting gallium compound, also exerted anti-OS activity as shown by a reduced clonogenic potential, increased apoptosis ratio, and an inhibitory effect on cell migration. Autophagy was believed to protect OS cells from KP46-mediated cell death, and the interaction between KP46 and an autophagy inhibitor was found to promote OS cell death (82). In contrast to the above studies indicating that activation of autophagy reduces OS cell death, Li et al. showed that induction of autophagy following treatment with triptolide (TPL), a diterpenoid epoxide extracted from *Tripterygium wilfordi*, exerted an anti-cancer effect. TPL treatment increased the rate of LC3II/ LC3I conversion and expression of beclin 1, followed later by a significant decrease in VEGF and HIF-1α. Furthermore, the autophagy inhibitor 3-MA clearly diminished this suppressive effect (83). Similarly, epigallocatechin gallate (EGCG), a polyphenol in green tea with antitumor bioactivity, triggered an autophagic inhibition response. Enhanced autophagy was observed by an increased ratio of LC3II/LC3I and decrease in P62 when OS was treated with Dox alone, but this treatment in combination with EGCG administration reversed the above effects and resulted in significant reductions in OS growth and viability (84).

## Dual Role of Autophagy in OS

A growing body of evidence has revealed that autophagy is associated with both cell survival and cell death in OS. On the one hand, autophagy is activated as a cytoprotective mechanism to provide tumor cells with nutrients and energy under conditions of starvation and hypoxia (85). In addition, autophagy endows OS cells with a resistant phenotype to ameliorate the cytotoxicity of drugs. On the other hand, autophagy plays an important role in OS suppression by inducing autophagic cell death, a kind of physiological cell death which is recognized as type II programmed cell death (86).

Several studies have shown that autophagy is capable of promoting the survival of OS cells. CYT997, a novel potent agent with anti-cancer activity, was reported to induce an enhancement of autophagy by elevating the numbers of GFP-LC3 puncta, formation of autophagosomes, and expression of LC3B-II and beclin 1. To determine the role of CYT997-mediated autophagy, the study authors combined CYT997 with the autophagy inhibitors 3-MA and CQ, or *Atg5*- and *Atg7*-targeted shRNA and determined the viability of treated OS cells. The results demonstrated that CYT997 plus the autophagy inhibitors yielded a dramatic anti-cancer effect, indicating that CYT997-induced autophagy can promote tumor growth (87). Another study investigating combretastatin A-4 (CA-4), a tubulin-depolymerizing agent with anti-tumor activity, revealed that CA-4 could activate autophagy as evidenced by a significant increase in the conversion from LC3-I to LC3-II and accumulation of autophagosomes. To explore whether CA-4-stimulated autophagy promoted or suppressed the growth of OS cells, the authors measured cell viability using a CCK8 assay with a combination of CA-4 and the autophagy inhibitor CQ. The results demonstrated that combined treatment markedly suppressed cell survival compared with CA-4 or CQ alone (88). The expression of GFRA1/GFRα1, the glycosylphosphatidylinositol-linked GDNF receptor α 1, was significantly induced by CDP at both the transcriptional and translational levels, whereas Dox and HD-MTX had little effect on its expression. Furthermore, a clear enhancement of LC3 puncta and formation of AVO were observed in GFRA1-positive OS cells after CDP treatment compared with GFRA1-deficient cells, suggesting that GFRA1 was required for CDP-mediated autophagy. More importantly, the authors found that the expression of GFRA1 reduced the sensibility of OS cells to CDP, showing that GFRA1-mediated autophagy in the presence of CDP decreased the apoptosis of OS cells (89).

Increasing evidence suggests that autophagy could promote cytotoxicity under certain circumstances or treatments. Activation of autophagy was observed in OS cells treated with tanshinone IIA (TIIA), a diterpenoid naphthoquinone extracted from the herb *Salvia miltiorrhiza*. TIIA treatment increased not only the amount of LC3B-II protein and LCB puncta, but also the formation of AVO, which is one of the characteristic features of autophagy. To further determine the mechanism of TIIA-induced autophagy, Yen et al. focused on BECN1 and SESEN2. They determined that knockdown of SESEN2, rather than inhibition of BECN1, attenuated the TIIA-mediated conversion from LC3-I to LC3-II. Furthermore, in an OS mouse model in which 143B OS cells were injected subcutaneously, increased levels of autophagy-related beclin 1, ATG5, ATG7, and class III PI3K were demonstrated after TIIA treatment, whereas the expression of anti-apoptotic BCL2 was decreased. Therefore, TIIA-induced autophagy revealed a pro-apoptotic function associated with the expression of SESEN2 (90). Another natural mixture, escin, which is derived from *hippocastanum* and has anti-cancer potential, could stimulate autophagy as confirmed by autophagosome formation measured by transmission electron microscopy (TEM) and elevated levels of LC3BII, ATG5, ATG12, and beclin 1. A moderate reduction in escin-induced cell death was observed for the combination of escin and 3-MA, whereas treatment in combination with another autophagy inhibitor, z-VAD-fmk, dramatically reduced cell death (91). It is clear that activation of autophagy augments cytotoxicity, resulting in an autophagic cell death.

## Application of Autophagy in OS Treatment

Substantial advances have been made in the treatment of OS owing to the use of neoadjuvant chemotherapy, which includes high-dose HD-MTX, Dox, CDP, and ifosfamide (IFO). Although neoadjuvant chemotherapy plays a crucial role in OS, the overall survival as remained at around 60% in the past 30 years. Drug resistance is a major factor that limits the survival rate of OS patients, and it is therefore critical to address this or develop new chemotherapeutic schemes. When cancer cells are treated with chemotherapy, activation of autophagy can be observed, and this chemotherapy-induced autophagy can promote resistance to the toxicity of drugs and eventually make cancer cells insensitive to the treatment. Several studies have shown that the combination of autophagy inhibitors and anti-tumor drugs can enhance the sensitivity of OS cells to the drugs and produce a greater anti-tumor effect. Apatinib, an inhibitor of the vascular endothelial growth factor receptor-2 (VEGFR2) tyrosine kinase (92), enhanced the expression of beclin 1 by inhibiting BCL2. Importantly, the combination of Apatinib and the autophagy inhibitor 3-MA, which can inhibit the initial formation of autophagosomes, significantly decreased cell viability and increased the ratio of apoptotic cells (93), suggesting that the use of 3-MA was at least one of the mechanisms resulting in enhanced cell death. Similarly, a study of celecoxib, a widely used anti-inflammatory drug, by Zhou et al. demonstrated that this drug can induce autophagy. This study also showed that pre-treatment with CQ or SAR405, a potent autophagy inhibitor of VPS18 and VPS34, markedly increased celecoxib-mediated apoptosis and the levels of cleaved PARP and cleaved caspase 3. Furthermore, the inhibition of autophagy caused by ATG5 knockdown enhanced the pro-apoptotic effect of celecoxib, indicating that targeting autophagy was beneficial for enhancing the celecoxib-induced cytotoxicity in OS (94). Consistent with the above findings, the authors concluded that targeting autophagy significantly increased the efficacy of chemotherapy-induced cell death.

In clinical settings, CDP is commonly used in OS chemotherapy (95). A direct effect of CDP on the induction of autophagy was observed, demonstrated by elevated levels of LC3-II. A synergistic effect of CDP and 3-MA on the viability of MG63 cells, which could further lead to cell death and apoptosis, was also noted (96). Based on the preclinical data mentioned above, we have an improved understanding of targeted autophagy as not only promoting tumor cell death but also sensitizing tumors to chemotherapies. Many clinical studies attempt to use autophagy inhibitor as a mean of regulating the response of patients to cancer therapies. Current research posted at ClinicalTrials.Gov is investigating the effects of CQ on breast cancer patients, and other clinical trials of targeted autophagy focus on different tumors, including colon cancer and glioblastoma (97). Similarly, a new phase I/II clinical trial (NCT03598595) is exploring whether gemcitabine and docetaxel in combination with hydroxychloroquine (HCQ) enhance the disease control rate in patients with recurrent/metastatic OS after 4 months of treatment. Furthermore, autophagy also has an anti-tumor property, and therefore, several related pharmacological compounds and strategies have been investigated in clinical studies (Table 1). Currently, rapamycin, a specific inhibitor of mTOR, has been the most extensively studied in OS clinical trials. More autophagy regulators need to be identified in the future, and their possible use as effective therapies against OS require further research.

**Table 1:** Clinical trials using autophagy inducers or inhibitors in OS treatment.

**Identifier**	**Intervention**	**Autophagy activation/inhibition**	**Phase**	**Title**
NCT03598595	docetaxel +gemcitabine+HCQ	Inhibition	I/II	Gemcitabine, docetaxel, and hydroxychloroquine in treating participants with recurrent or refractory osteosarcoma
NCT00093080	ridaforolimus	Activation	II	Study of AP23573/MK-8669(ridaforolimus), a mammalian target of rapamycin(mTOR) inhibitor, in participants with advanced sarcoma (MK-8669-018 AM1) (Completed)
NCT03213678	LY3023414	Activation	II	PI3K/mTOR inhibitor LY3023414 in treating patients with relapsed or refractory advanced solid tumors, non-Hodgkin lymphoma, or histiocytic disorders with TSC or PI3K/mTOR mutations (A pediatric match treatment trial)
NCT03190174	nivolumab+ nab-rapamycin	Activation	I/II	Nivolumab (Opdivo®) plus ABI-009(Nab-Rapamycin) for advanced sarcoma
NCT02429973	gemcitabine+ rapamycin	Activation	II	Trial with gemcitabine and rapamycin in second line of metastatic osteosarcoma (GEIS-29)

## Conclusions and Perspectives

Autophagy is a dynamic catabolic process that helps maintain cellular homeostasis by degrading non-essential organelles or proteins. It is a multi-step regulatory event that is activated in response to cellular stress, starvation, hypoxia, and cancer therapies. It plays roles in both promoting and suppressing tumor growth during different stages of tumor development and in various types of tumors. Here, we reviewed the functions of autophagy in OS, the related regulatory mechanisms, the different roles of autophagy in therapy-induced cell death, and the significance of targeted autophagy. Overall, the question remains whether autophagy promotes survival or causes cell death in OS. Future research is needed to clarify the level and function of autophagy in OS from the primary tumor to metastatic lesions, which will hopefully strengthen our understanding of the role of autophagy in the progression of OS and enable the development of effective targeted autophagy treatments.

A constructive step, which is vital and indispensable, is to examine the level of autophagy *in vivo* and in tumor tissue specimens from OS patients, and not only at the cellular level. Several studies have evaluated the correlation between the autophagy-related markers beclin 1 and LC3 and the prognosis of patients by evaluating cancer tissues by immunohistochemistry (IHC). This work led to the findings that beclin 1 predicts a favorable prognosis in gastric cancer (98) and breast cancer (99), whereas LC3 might predict a worse overall survival in breast cancer (100). Similar research has not been reported in OS; therefore, prospectively designed studies that evaluate the correlation between autophagy markers and the prognosis of OS patients are strongly recommended.

Currently, most research focuses on autophagy-related static indicators by detecting either the expression of LC3 or the formation of autophagosomes. These approaches may not precisely reflect the level of autophagy. The accumulation of autophagosomes may be caused either by increased degradation products after the activation of autophagy or by the blockage of an autophagic effect that prevents autophagosome clearance. Therefore, evaluation of the number of autophagosomes cannot fully demonstrate the activation of the overall autophagic system, suggesting that it is necessary to detect the formation of autophagic flux, a dynamic process, to accurately determine the level of autophagy.

In addition, pulmonary metastasis is the main cause of the low event-free survival and increased rate of death of OS patients. Inhibiting lung metastasis or improving sensitivity of lung metastatic lesions to therapies remains a formidable challenge. At this time, there are few reports on the relationship between drug insensitivity and autophagy of metastatic lung lesions in OS. Additional research is needed to determine the role autophagy plays in drug resistance and the formation of pulmonary metastases in this disease. Altogether, with the advancements in our understanding of autophagy and related regulatory mechanisms in OS, we expect that there will be more specific findings of autophagy modulators that could enhance the efficacy of anti-OS therapies.

## Author Contributions

JN and WW wrote most of the manuscript. ZZ made the figure and table. WG revised and amended the manuscript. TY conceived, thoroughly revised, and amended the manuscript.

### Conflict of Interest

The authors declare that the research was conducted in the absence of any commercial or financial relationships that could be construed as a potential conflict of interest.

## Abbreviations

ATG: autophagy-related
PI-3KIII: class III phosphatidylinositol-3 kinase
Vps34: vesicular protein sorting 34
PE: phosphatidylethanolamine
LC3: microtubule-associated protein 1 light chain 3
p62/SQSTMl: equestosome 1
mTOR: mammalian target of rapamycin
PI3K: phosphoinositide-3-kinase
AKT: protein kinase B
ULK1: UNC-51-like kinase 1
DPT: deoxypodophyllotoxin
3-MA: 3-methyladeine
Hsp90: heat shock protein 90
GA: geldanamycin
ROS: reactive oxygen species
JNK: C-Jun N-terminal kinase
ATO: arsenic trioxide
As_2_S_2_: arsenic sulfide
NAC: N-acetyl-L-cysteine
AVO: acidic vesicular organelle
AG: andrographolide
IP3K2: inositol 1,4,5- trisphosphate kinase 2
GBM: glioblastoma
HIF1: hypoxia-inducible factor 1
BNIP3: adenovirus E1B 19 kDa interacting protein 3
CQ: chloroquine
WT1: wilm's tumor suppressor1
Gas1: arrest-specific 1
ERK: mitogen-activated protein kinase
TPL: triptolide
EGCG: epigallocatechin gallate
CA-4: combretastatin A-4
CCK8: cell counting kit−8
TIIA: tanshinone IIA
TEM: transmission electron microscopy
HD-MTX: methotrexate
Dox: doxorubicin
CDP: cisplatin
IFO: ifosfamide
VEGFR2: vascular endothelial growth factor receptor-2
HCQ: hydroxychloroquine
IHC: immunohistochemistry.
